# Supplementary material for: Melanocortin-4 receptor antagonist TCMCB07 alleviates chemotherapy-induced anorexia and weight loss in rats
Source: J Clin Invest. 2024 Nov 7;135(1):e181305. doi: 10.1172/JCI181305 (PMC11684807; doi:10.1172/JCI181305)
Supplement: Supplemental data [file jci-135-181305-s179.pdf]

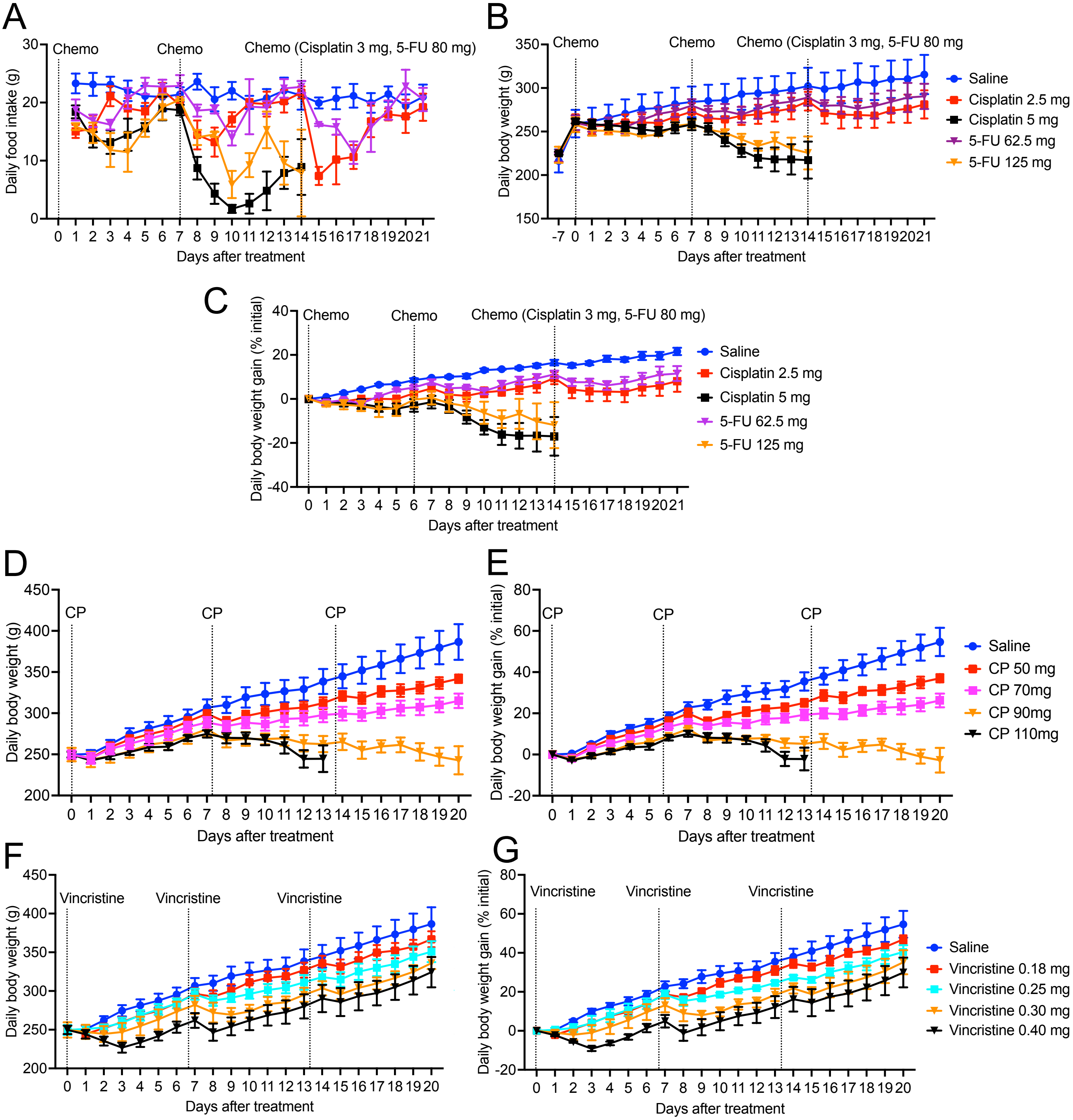

Supplemental Figure 1. Related to Figure 1

Chemotherapy dosing regimen selection. (**A**) Daily food intake, (**B**) Daily body weight, and (**C**) Daily body weight gain (% initial body weight), following each cycle of chemotherapy with cisplatin administered at doses of 2.5 or 5 mg/kg, or with 5-FU at doses of 62.5 or 125 mg/kg. During the third cycle of chemotherapy, the high-dose of cisplatin and 5-FU were discontinued due to mortality and severe deterioration in condition. The low dose of cisplatin was adjusted from 2.5 to 3 mg/kg, and the low dose of 5-FU was adjusted from 62.5 to 80 mg/kg. All data in (**A**-**C**) were expressed as mean ± SEM for each group. *n* = 4. (**D**) and (**F**) Daily body weight, and (**E**) and (**G**) Daily body weight gain (% initial body weight), following each cycle of chemotherapy with cyclophosphamide administered at doses of 50, 70, 90, or 110 mg/kg, or with vincristine at doses of 0.18, 0.25, 0.30, or 0.40 mg/kg. All data in (**D**-**G**) were expressed as mean ± SEM for each group. n = 4.

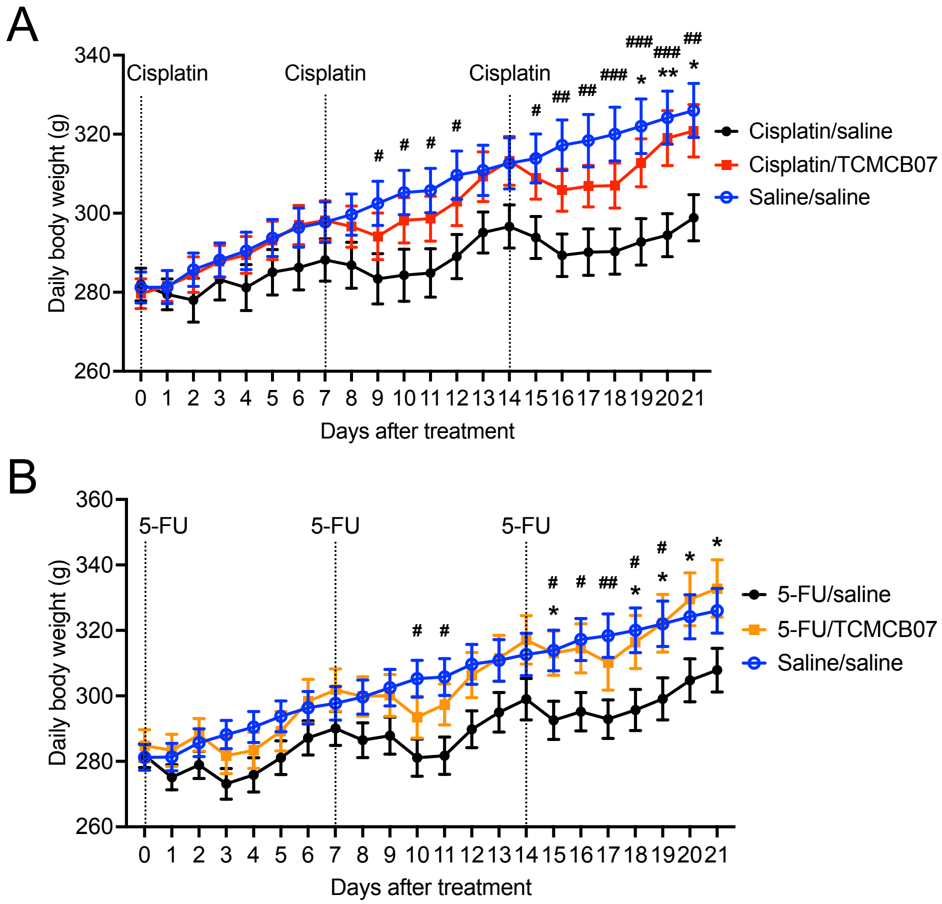

Supplemental Figure 2. Related to Figure 4

TCMCB07 treatment maintains body weight throughout multiple cycles of cisplatin or 5-fluorouracial chemotherapy. Daily body weight after (**A**) cisplatin and (**B**) 5-fluorourcial (5-FU) chemotherapy and TCMCB07 treatment. All data in (**A**) and (**B**) were expressed as mean ± SEM for each group. *n* = 10-12. *: Chemotherapy/saline vs Chemotherapy/TCMCB07, #: Chemotherapy/saline vs Saline/saline. *, #, *P* < 0.05; **, ##, *P* < 0.01; ###, *P* < 0.001; Two-way ANOVA.

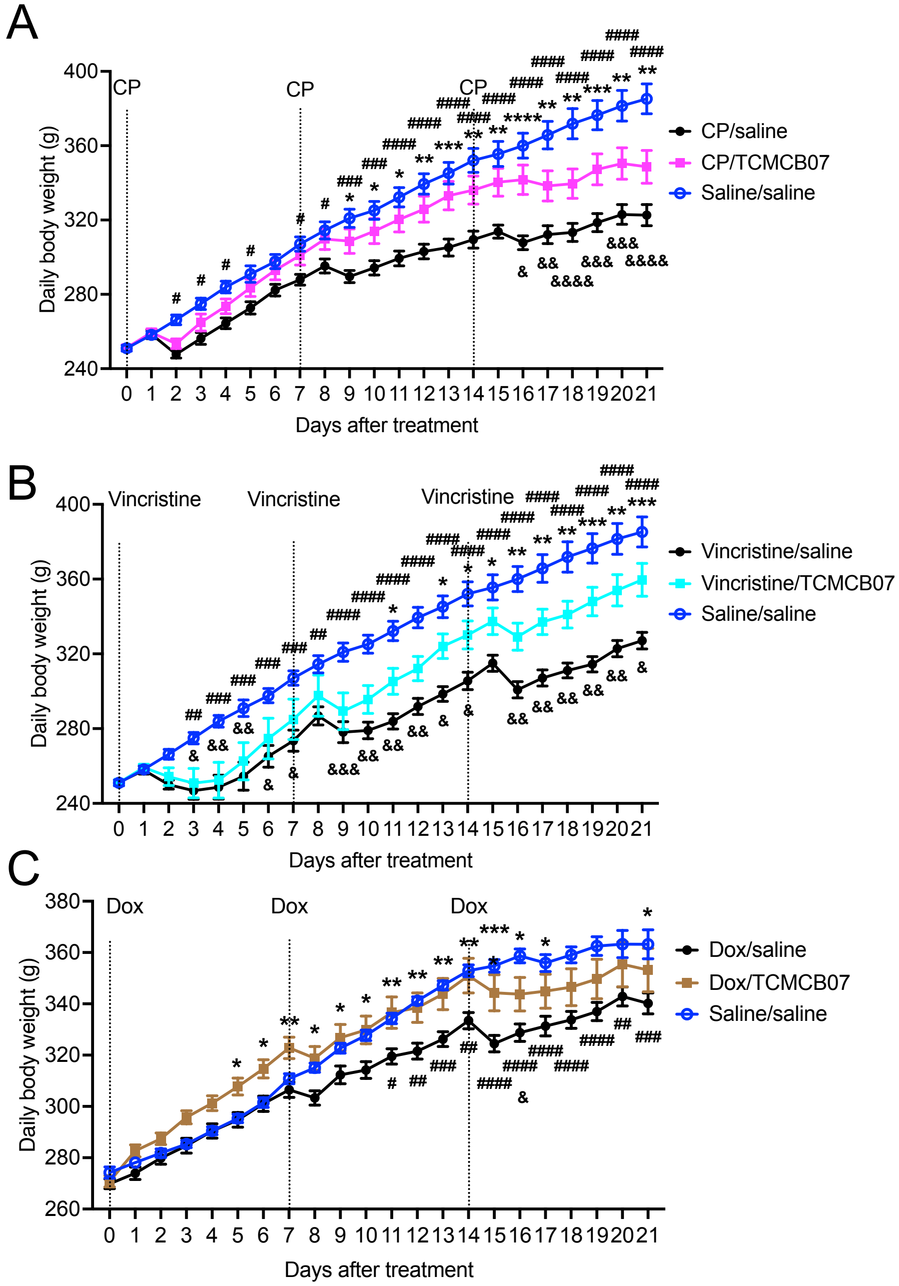

Supplemental Figure 3. Related to Figure 5

TCMCB07 treatment maintains body weight throughout multiple cycles of cyclophosphamide, vincristine, or doxorubicin chemotherapy. Daily body weight after (**A**) cyclophosphamide, (**B**) vincristine, or (**D**) doxorubicin chemotherapy and TCMCB07 treatment. All data in (**A**-**C**) were expressed as mean ± SEM for each group. *n* = 10-12. *: Chemotherapy/saline vs Chemotherapy/TCMCB07, #: Chemotherapy/saline vs Saline/saline, &: Chemotherapy/TCMCB07 vs Saline/saline. *, #, &, *P* < 0.05; **, ##, &&, *P* < 0.01; ***, ###, &&&, *P* < 0.001; ****, ####, &&&&, *P* < 0.0001; Two-way ANOVA.

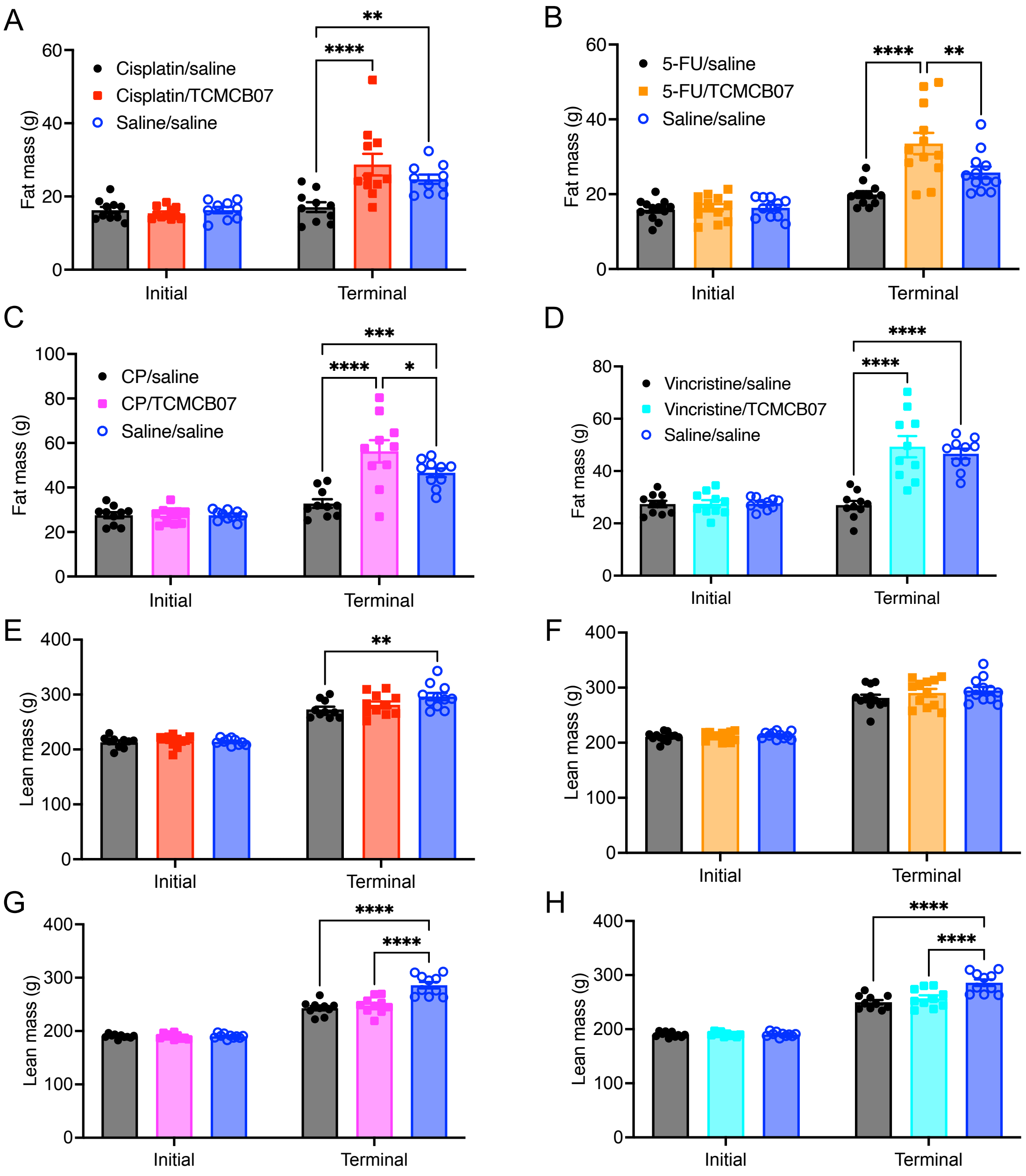

Supplemental Figure 4. Related to Figure 6

TCMCB07 treatment attenuates chemotherapy-induced fat and lean mass loss. Fat mass and lean mass after chemotherapy of cisplatin (**A**) and (**E**), 5-FU (**B**) and (**F**), cyclophosphamide (**C**) and (**G**), or vincristine (**D**) and (**H**) and TCMCB07 treatment. All data in (**A-H**) were expressed with each dot representing one sample. $n = 10\text{-}12$. *$P < 0.05$; **$P < 0.01$; ***$P < 0.001$; ****$P < 0.0001$; Two-way ANOVA.

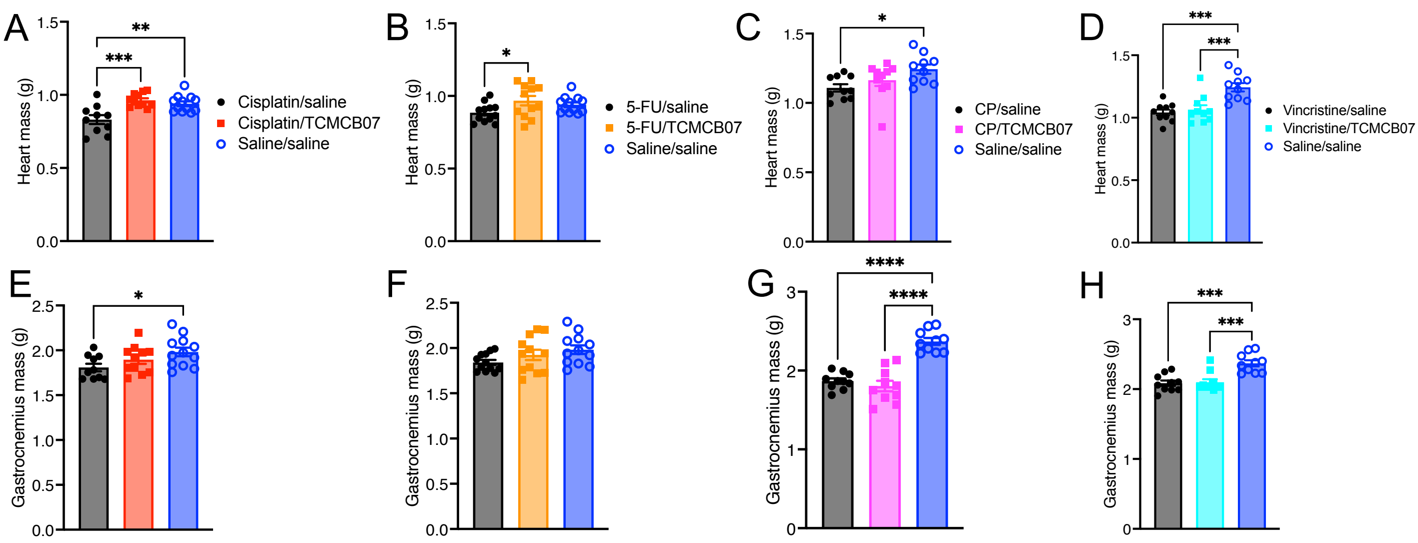

Supplemental Figure 5. Related to Figure 7

TCMCB07 treatment protects heart tissue during multiple cycles of chemotherapy. Heart and gastrocnemius mass after chemotherapy of cisplatin (**A**) and (**E**), 5-FU (**B**) and (**F**), cyclophosphamide (**C**) and (**G**), or vincristine (**D**) and (**H**) and TCMCB07 treatment. All data in (**A-H**) were expressed with each dot representing one sample. *n* = 10-12. **P* < 0.05; ***P* < 0.01; ****P* < 0.001; *****P* < 0.0001; One-way ANOVA.

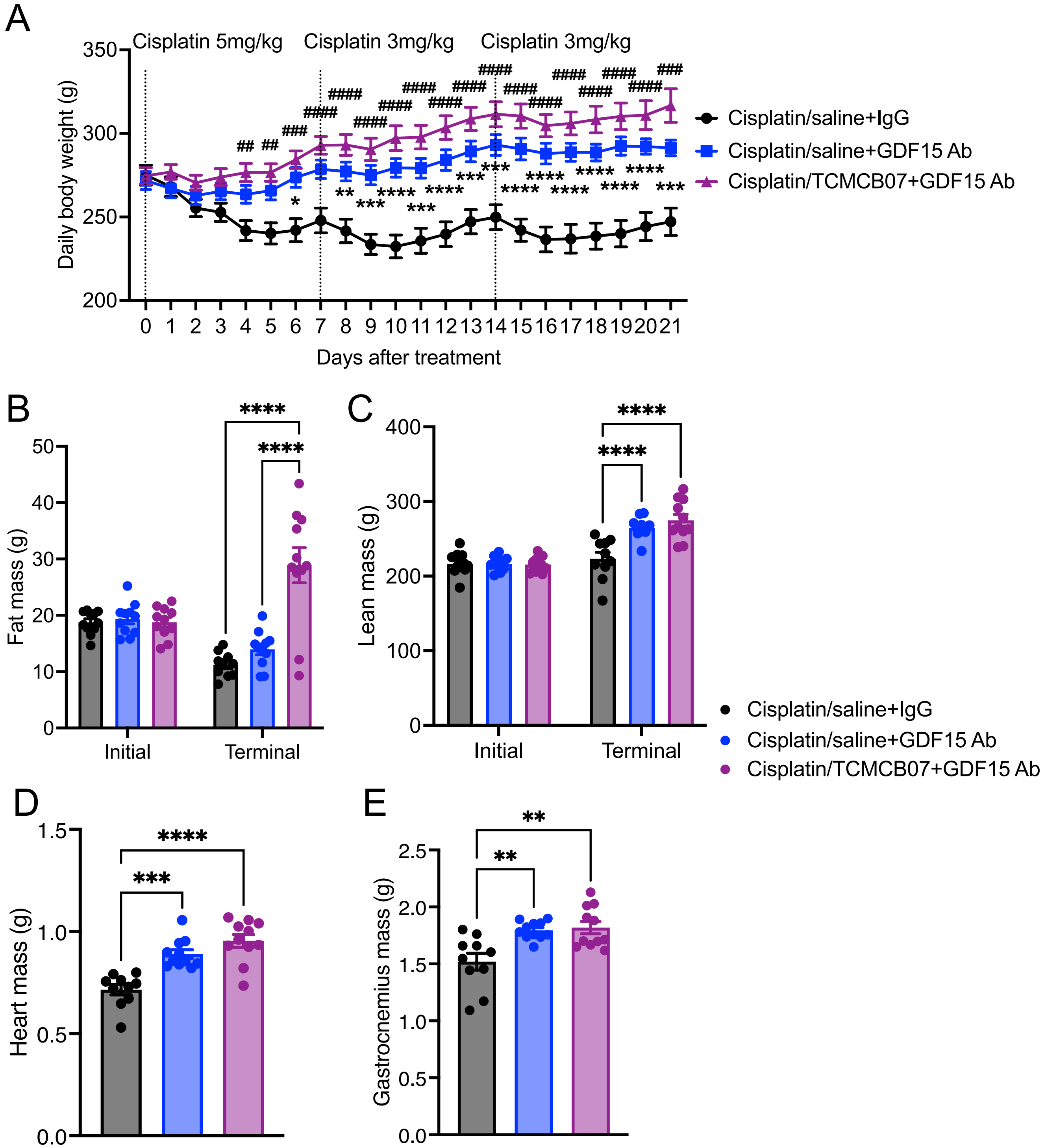

Supplemental Figure 6. Related to Figure 10

Combination therapy of TCMCB07 + GDF15 antibody improves effectiveness in maintaining body and tissue mass during chemotherapy. (**A**) Daily body weight, (**B**) Fat mass, (**C**) Lean mass, (**D**) Heart mass, and (**E**) Gastrocnemius mass after treatment of cisplatin+saline+IgG, cisplatin+saline+GDF15 antibody (Ab), or cisplatin+TCMCB07+GDF15 Ab. All data in (**A**) were expressed as mean ± SEM for each group. All data in (**B**-**E**) were expressed with each dot representing one sample. n = 10-11. *, $P < 0.05$; **, ##, $P < 0.01$; ***, ###, $P < 0.001$; ****, ####, $P < 0.0001$; Two-way ANOVA (**A**-**C**), One-way ANOVA (**D**) and (**E**).

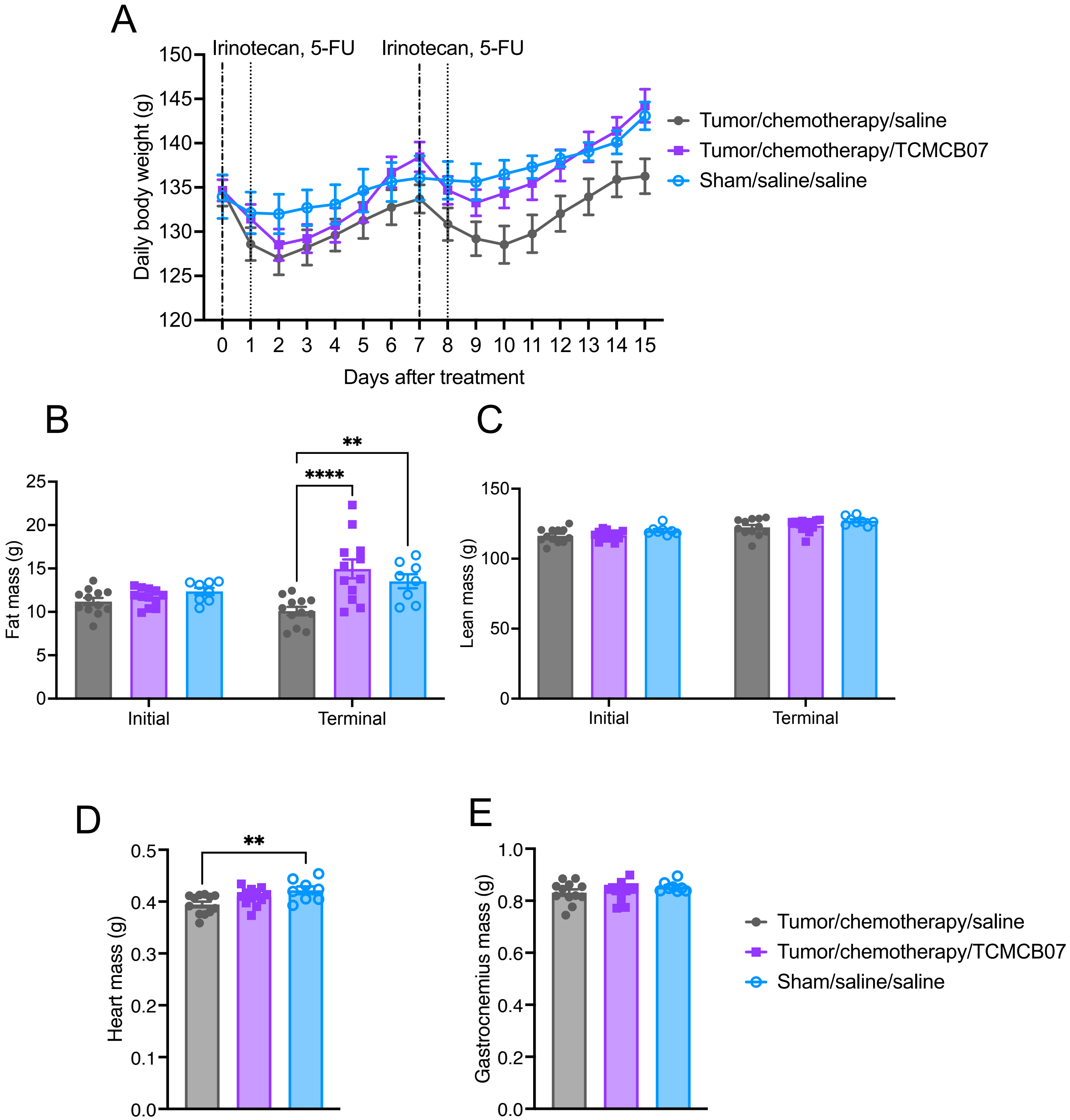

Supplemental Figure 7. Related to Figure 12

TCMCB07 treatment mitigates anorexia and weight loss in rats with Ward colorectal tumor following combination chemotherapy. (**A**) Daily body weight, (**B**) Fat mass, (**C**) Lean mass, (**D**) Heart mass, and (**E**) Gastrocnemius mass after combination chemotherapy of irinotecan and 5-FU and TCMCB07 treatment. All data in (**A**) were expressed as mean ± SEM for each group, and all data in (**B-E**) were expressed with each dot representing one sample. *n* = 8-12. **, *P* < 0.01; ****, *P* < 0.0001; Two-way ANOVA (**A-C**), One-way ANOVA (**D**) and (**E**).

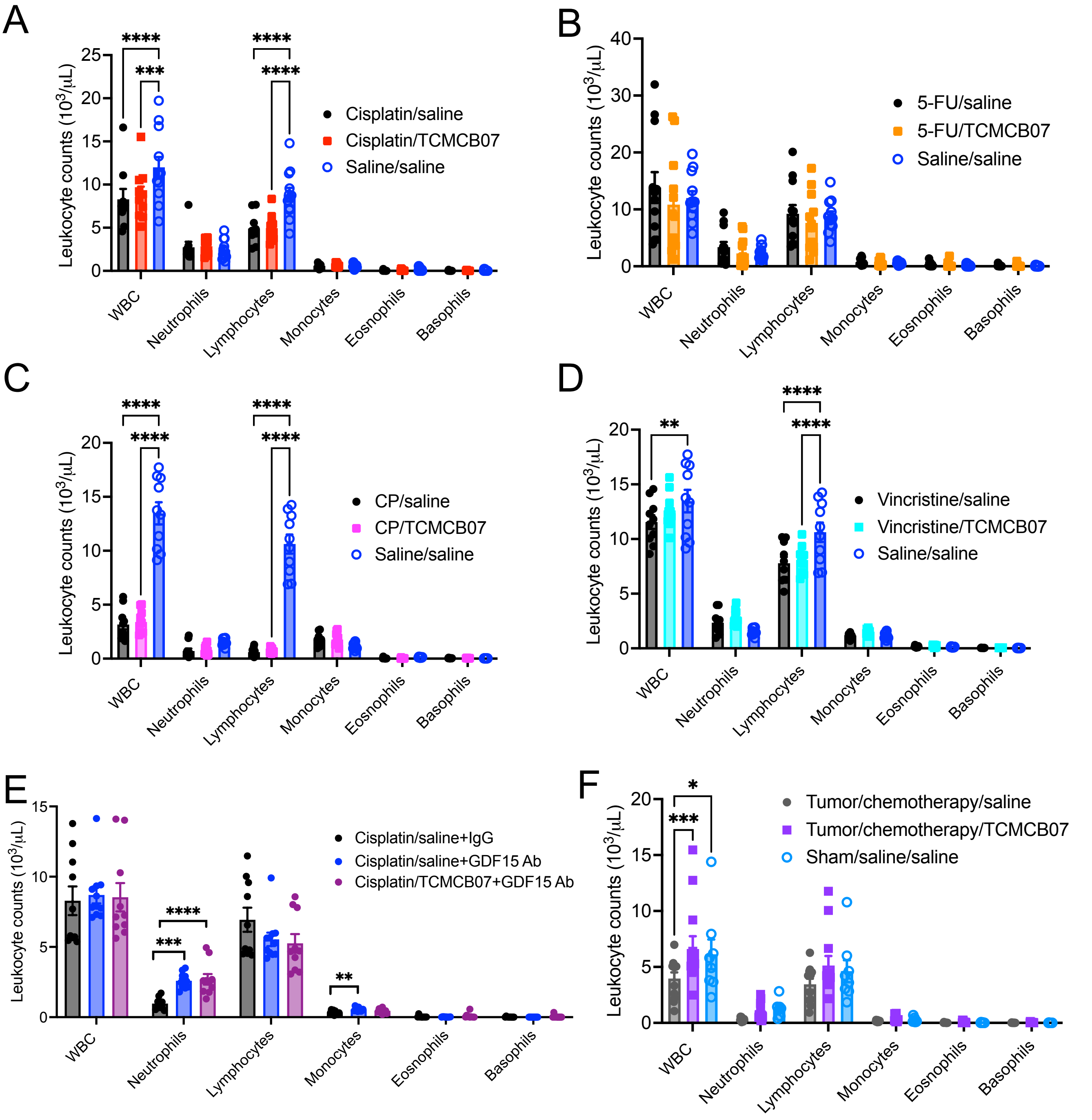

Supplemental Figure 8. Related to Table 1

Leukocyte counts in rat blood. Blood samples were collected from experiments: (**A**) Cisplatin/TCMCB07, (**B**) 5-FU/TCMCB07, (**C**) Cyclophosphamide/TCMCB07, (**D**) Vincristine/TCMCB07, (**E**) Combination therapy of cisplatin+TCMCB07+GDF15 antibody, and (**F**) Irinotecan and 5-FU combination chemotherapy and TCMCB07 treatment in rats with Ward colorectal tumor. All data in (**A**-**F**) were expressed with each dot representing one sample. *n* = 8-12. *, $P < 0.05$; **, $P < 0.01$; ***, $P < 0.001$; ****, $P < 0.0001$; Two-way ANOVA.
